# Aberrant X chromosome skewing and acquired clonal hematopoiesis in adult-onset common variable immunodeficiency

**DOI:** 10.1172/jci.insight.127614

**Published:** 2019-07-25

**Authors:** Gabriel K. Wong, Sara Barmettler, James M. Heather, David Millar, Sarah A. Penny, Aarnoud Huissoon, Alex Richter, Mark Cobbold

**Affiliations:** 1 Institute of Immunology and Immunotherapy, Medical School, University of Birmingham, Edgbaston, United Kingdom.; 2 Allergy and Clinical Immunology Unit, Division of Rheumatology, Allergy and Immunology, Massachusetts General Hospital, Boston, Massachusetts, USA.; 3 Massachusetts General Hospital, Cancer Center and Department of Medicine, Harvard Medical School, Boston, Massachusetts, USA.; 4 West Midlands Immunodeficiency Centre, Birmingham Heartlands Hospital, Birmingham, United Kingdom.

**Keywords:** Hematology, Immunology, Adaptive immunity, Cancer

## Abstract

Advances in genomic medicine have elucidated an increasing number of genetic etiologies for patients with common variable immunodeficiency (CVID). However, there is heterogeneity in clinical and immunophenotypic presentations and a limited understanding of the underlying pathophysiology of many cases. The primary defects in CVID may extend beyond the adaptive immune system, and the combined defect in both the myeloid and lymphoid compartments suggests the mechanism may involve bone marrow output and earlier progenitors. Using the methylation profile of the human androgen receptor (AR) gene as a surrogate epigenetic marker for bone marrow clonality, we examined the hematopoietic compartments of patients with CVID. Our data show that clonal hematopoiesis is common among patients with adult-onset CVID who do not have associated noninfectious complications. Nonblood tissues did not show a skewed AR methylation status, supporting a model of an acquired clonal hematopoietic event. Attenuation of memory B cell differentiation into long-lived plasma cells (CD20^–^CD27^+^CD38^+^CD138^+^) was associated with marked changes in the postdifferentiation methylation profile, demonstrating the functional consequence of clonal hematopoiesis on humoral immunity in these patients. This study sheds light on a potential etiology of a subset of patients with CVID, and the findings suggest that it is a stage of an acquired lymphocyte maturation disorder.

## Abstract 2

Clonal hematopoiesis is common among a subset of patients with common variable immunodeficiency (CVID), suggesting that CVID may be a stage of lymphoid dysplasia.

## Introduction

Primary immunodeficiencies (PIDs) are classically described as inherited errors of the immune system (1). With advances in molecular biology, numerous monogenic mutations with functional consequences have now been identified as the causes for these disorders (2, 3). Although many of these disorders are inherited and are observed in early childhood, there are several examples of PIDs in which mutations affect noncanonical pathways of immune responses or present later in life because of incomplete or variable penetrance. For example, deficiencies in the terminal complement pathway (C5–9) commonly present in late adolescence with *Neisseria meningitis* infections (4, 5). There are also cases of patients with genetic mutations in which the fulminant presentation has been triggered by an infectious complication, such as in late-onset hemophagocytic lymphohistiocytosis (ref. 6), hypomorphic variants presenting later in life such as with RAG mutations (7), or a potential accumulation of detrimental variants in immunological pathways (8).

Relatively little is known, however, about other late-onset PIDs, such as common variable immunodeficiency (CVID), with which almost two-thirds of patients present in adulthood (1). Although an increasing number of monogenetic mutations have been found in adult patients with CVID — including CD19, CD20, CD21, CD27, CD81, CTLA4, ICOS, NFκB1, NFκB2, and PI3KD (3, 9–17) — up to 70% of patients still lack an identifiable molecular diagnosis. Large cohort studies have shown that the onset of disease peaks around the third decade, though it may occur at any age (18–20). However, accurate epidemiology data are difficult to ascertain because diagnostic delay in CVID continues to be a major problem for patients, given the variability in presentations, despite decades of efforts to raise awareness among physicians (21). Furthermore, the continual evolution of the definition and diagnostic criteria for CVID makes comparison with historical cohorts difficult (22). The majority of adult-onset CVID cases remain enigmatic, lacking clear disease mechanisms and limiting our understanding of the etiology of this group.

Hypogammaglobulinemia is the central feature of CVID, with a relative or complete loss of plasma cells in the bone marrow and mucosal tissues (23, 24). A variable degree of hypogammaglobulinemia across different isotypes may be observed in patients, though significant reductions in IgG as well as low IgA or IgM are required to make the diagnosis (25). As a functional consequence, there are recurrent infections with encapsulated organisms, such as *Streptococcus pneumoniae*, *Haemophilus influenzae*, and *Moraxella catarrhalis* (26). Furthermore, patients with CVID may suffer from a range of noninfectious complications, including splenomegaly, lymphoproliferation, nodular regenerative hyperplasia of the liver, chronic enteropathy, granulomatous lymphocytic interstitial lung disease, skin infiltration, autoimmune cytopenia, and other autoimmune diseases (21). Prior studies have shown that patients with noninfectious complications have increased mortality, with 1 study reporting a risk of death that was 11 times higher for patients with CVID with 1 of more of the noninfectious complications as compared with those with infectious complications alone (HR 10.96; *P* < 0.0001; ref. 27). In the same study, females were shown to have shorter survival and greater risk of lymphoma. Stratification of patients by clinical presentation has been challenging given the significant overlap between complications and presentations; however, there have been recent advances regarding clustering of noninfectious complications and immunophenotypes in CVID (28).

Similarly, heterogeneity can be observed in immune parameters, even in patients sharing disease-associated polymorphisms (29, 30). Studies on B lymphocytes of CVID showed diversity in B cell count, memory B cell frequencies, proliferation, in vitro immunoglobulin production, and somatic hypermutation (31–33). However, B cells are precursors of plasma cells, which are antibody-secreting cells that reside within highly specialized niche environments. Functional studies on plasma cells are difficult to carry out, but histological examination has often revealed their paucity in patients with CVID (34, 35). Increasingly, defects in T lymphocytes are being recognized in CVID, with studies demonstrating variable degrees of lymphopenia, T cell exhaustion, impaired T regulatory cells, clonal expansion, and cytokine production (36, 37). Although the clinical and immunological heterogeneity may be related to the historical inclusion of numerous rare monogenetic mutations under the diagnostic bracket of CVID, it is also likely that heterogeneity is inherent to the pathophysiology of the disorder.

Given the late onset of the disorder and the absence of a known family history for many patients, CVID frequently behaves more like an acquired disorder. In this study, we demonstrate the presence of clonal hematopoiesis in a substantial proportion of female patients with CVID via an abnormal epigenetic signature, skewed X chromosome inactivation, with functional consequences in long-lived plasma cell generation. We postulate that CVID may represent an acquired lymphocyte maturation disorder in a susceptible host, which potentially explains the late onset and heterogeneous nature of the disease.

## Results

### X chromosome skewing demonstrated in patients with CVID.

Analysis of the methylation profile of the androgen receptor (AR) gene (located on the X chromosome, used as a surrogate marker for bone marrow clonality) was performed using genomic DNA that had been extracted from the whole blood of 23 female patients with CVID and 22 age-matched healthy females to examine bone marrow clonality. Participant characteristics are shown in Table 1.

**Figure 1:**
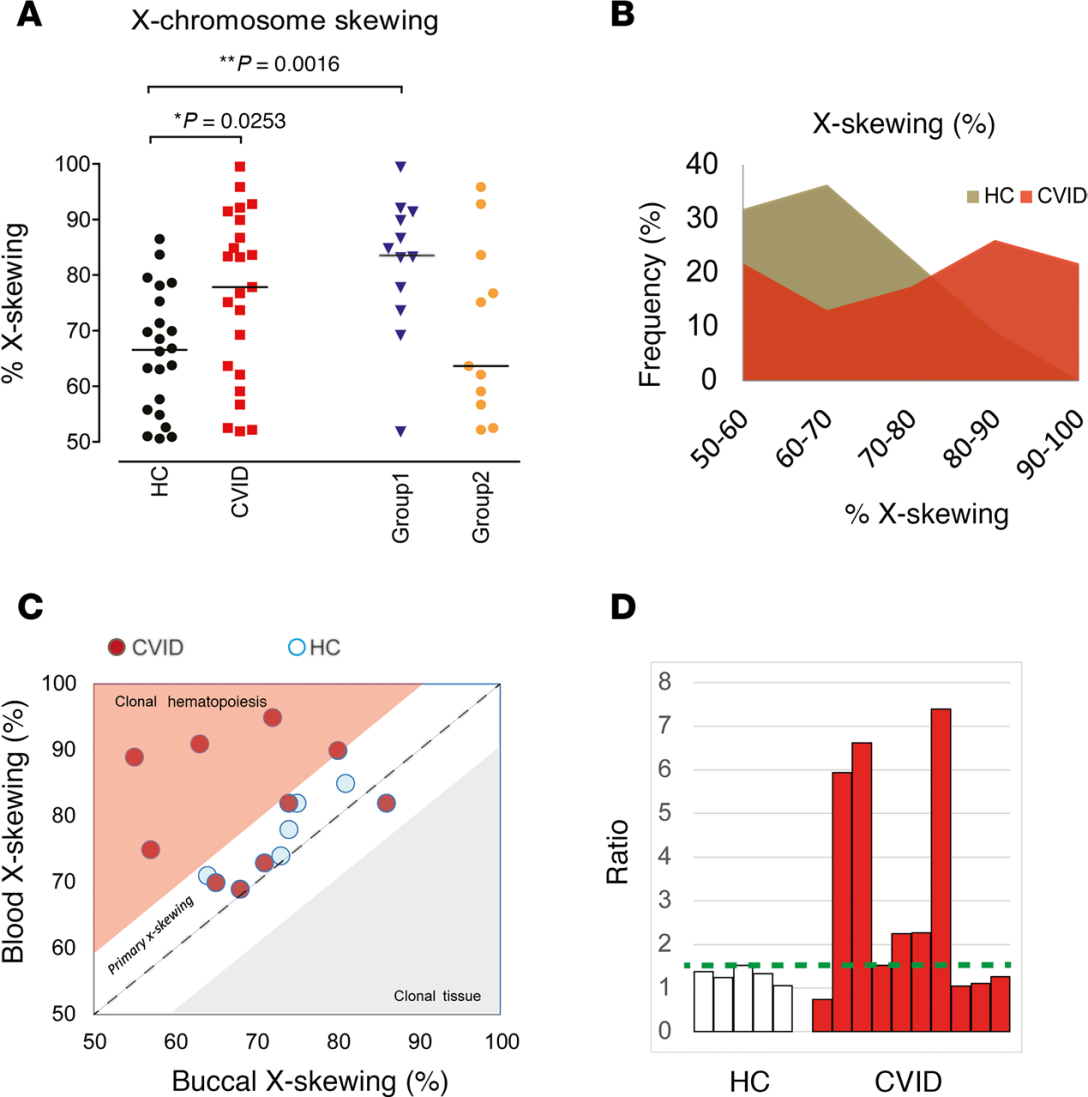
Exaggerated X chromosome skewing demonstrated in CVID group 1 patients (patients with infectious complications only) by HUMARA assay. X chromosome skewing of whole-blood DNA was determined using the HUMARA assay. X-skewing was corrected by the matching undigested control. (**A**) Results of female healthy controls (HCs) (*n* = 22), CVID patients (*n* = 23), group 1 patients (*n* = 12), and group 2 patients (*n* = 11) are shown. The horizontal lines represent the median, and statistical differences are highlighted (Mann-Whitney *U* test). (**B**) The frequencies of X-skewing of HCs and patients with CVID are shown, demonstrating a right shift within the CVID cohort. Comparison of buccal and blood X-skewing suggested acquired clonal hematopoiesis in patients with CVID but primary X-skewing in healthy donors. (**C**) X-skewing by the HUMARA assay was compared between matching blood and buccal samples of healthy donors (*n* = 5, light blue circles) and patients with CVID (*n* = 10, red circles) who had previously demonstrated high levels of X-skewing in their whole-blood analysis. The dotted blue line represents a theoretical 1:1 ratio. Dots in the white zone are considered to have primary X-skewing, while dots in the pink zone are considered to have clonal hematopoiesis. (**D**) The X-skewing ratios in the blood were adjusted to the expected ratios observed in the buccal sample and depicted as a bar chart (HCs, white; CVID, red). The highest HC blood/buccal ratio (1.6) is marked by the green dotted line.

Comparison of the X chromosome skewing ratios (determined by the assay) of patients with CVID and controls revealed that only a minority of healthy donors (2 of 22 or 9.1%) exhibited moderate to high X-skewing (>80%), consistent with published literature (38). In contrast, moderate to severe X-skewing was seen in 11 of 23 patients with CVID (47.8%). Interestingly, exaggerated skewing was predominately seen in group 1 patients (those with only infectious complications, with or without bronchiectasis). X-skewing in group 2 patients (those who had noninfectious complications, such as polyclonal lymphoproliferation, chronic enteropathy, interstitial lung disease, autoimmunity, or combinations of these) did not significantly differ from that of healthy donors. Despite being age matched, the greater prevalence of patients with CVID expressing high X-skewing supports the presence of clonality within the blood compartment (Figure 1, A and B).

### Clonality observed in the blood but not in tissues of patients with CVID.

Given that these data suggested that X-skewing is common among group 1 patients with CVID (who did not have noninfectious complications), we sought to test whether this clonal signature is unique to the hematopoietic compartment. Buccal samples were used as surrogate germline (nonblood) tissues for comparison. Matching buccal and blood samples from 5 healthy donors and 10 patients with CVID (CVID01, CVID03, CVID04, CVID05, CVID07, CVID10, CVID11, CVID13, CVID19, and CVID23) demonstrating a higher degree of X-skewing were analyzed, and the skewing ratios in the blood were plotted against the ratios observed in the matching buccal sample (Figure 1, C and D). All 5 healthy donors exhibited a very similar level of X-skewing in their blood and buccal tissues, consistent with comparable random X-inactivation during both embryogenesis and hematopoiesis, with no selective differences engendered by either chromosome being activated. In contrast, 50% of patients with CVID demonstrated a discrepancy in blood versus buccal X-skewing. Adjusted X-skewing ratios showed that the dominant allele in CVID might be as high as 7 times the frequency expected from autologous nonhematopoietic tissues. A similar clinical presentation can be seen in these patients (Supplemental Table 1; supplemental material available online with this article; https://doi.org/10.1172/jci.insight.127614DS1). This suggests that the clonal epigenetic signature of X-skewing was unique within the hematopoietic system in some patients with CVID and that the defect was unlikely to occur at the germline level. This supports the hypothesis that some cases of CVID may be acquired through genetic or epigenetic changes during life.

### Clonal penetrance in myeloid and lymphoid cells.

**Figure 2:**
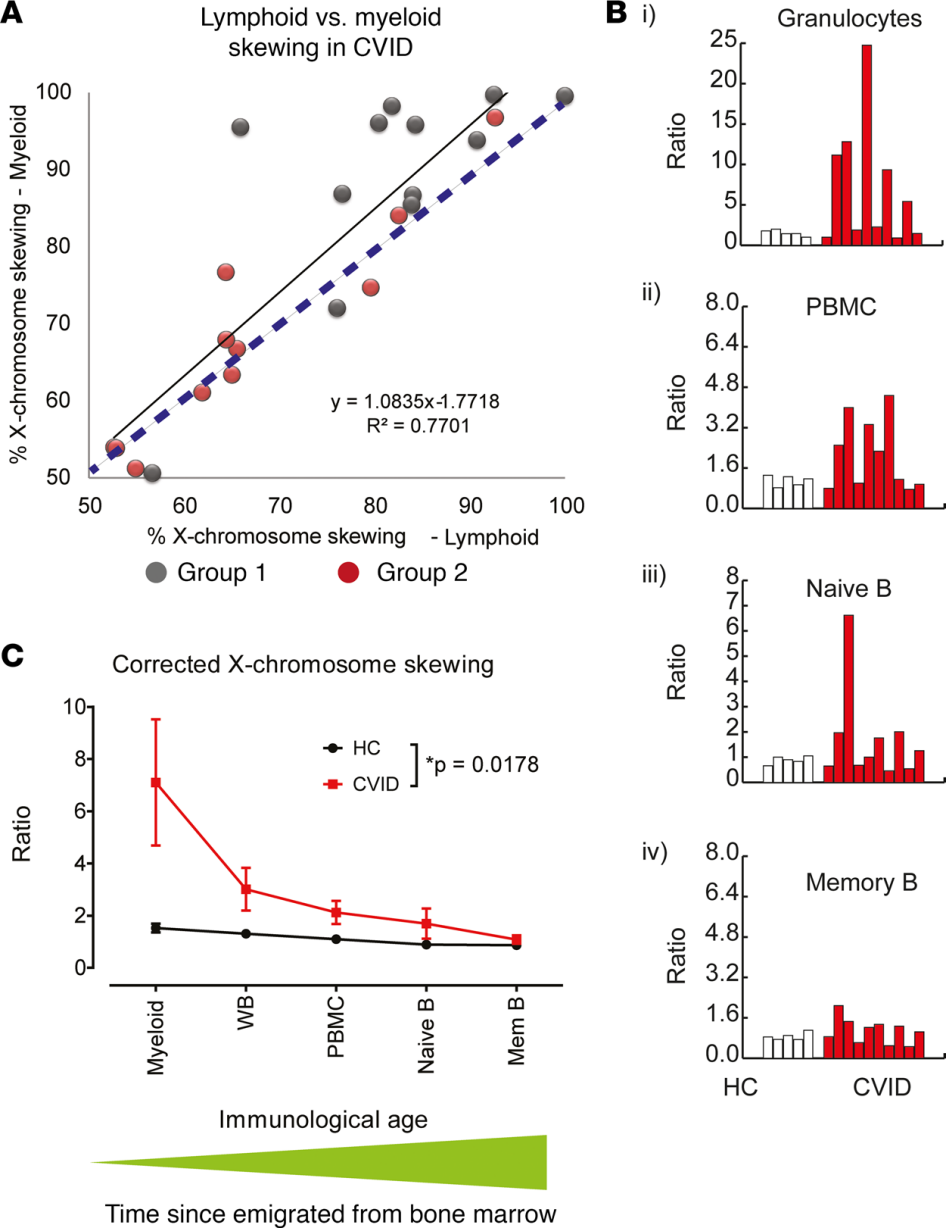
The assessment of clonal hematopoiesis in hematological subsets of various age. X-skewing of myeloid cells (represented here by granulocytes) and lymphoid cells (represented here by PBMCs) of patients with CVID (*n* = 23; group 1, *n* = 12, gray; group 2, *n* = 11, red) were compared. (**A**) Linear regression (black line) and *r^2^* are shown, while the blue dashed line represents a theoretical 1:1 ratio. (**B**) Using buccal X-skewing as a baseline, the ratio of allele 1 and allele 2 in granulocytes, PBMCs, naive B cells (CD27^–^), and memory B cells (CD27^+^) were adjusted to the expected value obtained from the matching buccal tissue (HCs, *n* = 5, white; CVID, *n* = 10, red). (**C**) Adjusted X-skewing ratios are plotted against immunological time, with granulocytes representing the most recent output from bone marrow and memory B cells representing historical output. Mean and SEM are shown (HCs, *n* = 5; CVID, *n* = 10; 2-way ANOVA). Whole blood (WB).

To evaluate the level at which clonal proliferation might have occurred in CVID, peripheral blood was separated into myeloid granulocytes and peripheral blood mononuclear cells (PBMCs, which are primarily of lymphoid origin). Abnormal X-skewing was detectable in both the myeloid and lymphoid compartments but was much greater in the myeloid compartment, suggesting that clonality was imprinted at the level of an early progenitor before differentiation into distinct lineages, i.e., clonal hematopoiesis (Figure 2A). To further examine how altered clonality penetrates through various hematopoietic lineages, naive and memory B cells were analyzed. The X-skewing of granulocytes, PBMCs, naive B cells, and memory B cells was adjusted according to their matching buccal samples (Figure 2B). There was little change in the skewing ratio seen in all leukocyte subsets of healthy donors when compared with the baseline buccal X-skewing. However, clonality was not uniformly distributed across all CVID patient leukocyte subsets, with the greatest effect seen in granulocytes, which represent the most recent bone marrow emigrants. Furthermore, clonality decreased in a stepwise fashion with populations containing a greater proportion of long-lived cells (Figure 2C), supporting the presence of an acquired clonal defect occurring in a progenitor cell in CVID.

### Partial failure in long-lived plasma cell differentiation in patients with CVID.

**Figure 3:**
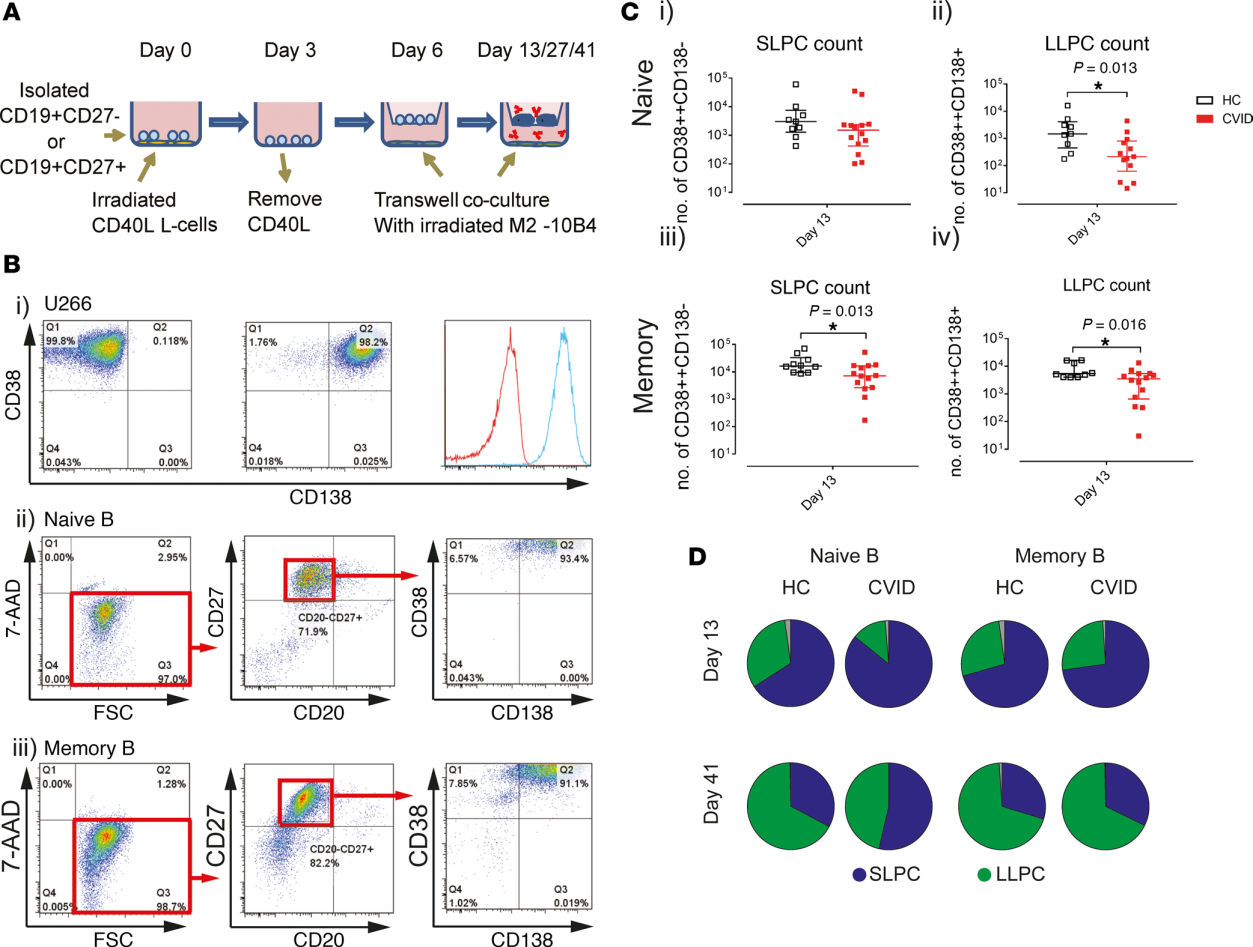
Numerical deficit in long-lived plasma cell generation by patients with CVID. Naive (CD27^–^) and memory (CD27^+^) B cells were enriched by magnetic bead isolation and terminally differentiated into plasma cells using a 3-step culture system. B cells were initially activated by CD40L-expressing L cells supplemented with IL-2, IL-21, and anti–IgA/M/G F(ab′)_2_ and then transferred to a Transwell system supported by murine bone marrow fibroblasts (M2-10B4) on day 6. The wells were harvested on days 13 and 41 for flow cytometric analysis. Short-lived plasma cells (SLPCs: 7-AAD^–^CD20^–^CD27^+^CD38^+^CD138^–^) and long-lived plasma cells (LLPCs: 7-AAD^–^CD20^–^CD27^+^CD38^+^CD138^+^) were enumerated using counting beads. (**A**) Schematic diagram of LLPC culture. (**B**) The gating strategy is shown. A U266 myeloma cell line was used to optimize CD38 and CD138 gating. Events within the upper-left quadrant of the far-right gate were considered SLPCs, whereas events within the upper-right quadrant were considered LLPCs. Examples of both naive and memory B cell differentiation of a healthy donor at day 13. (**C**) SLPC and LLPC counts generated from naive and memory cultures at day 13 of healthy donors (*n* = 10) and patients with CVID (*n* = 14). Median and interquartile range are represented by the height and error bar, respectively. Mann-Whitney *U* test was used. Statistical significance is indicated as *P < 0.05. (**D**) The proportions of SLPCs (blue) and LLPCs (green) within naive and memory cultures at day 13 and day 41 are shown in a series of pie charts.

The failure of antibody production can be incomplete in a subset of patients with CVID. We therefore hypothesized that clonal hematopoiesis may affect B cell functions but that residual antibody production is retained by B cells that were generated before the clonal event, preventing complete collapse of the antibody-producing machinery. To test this hypothesis, short-lived plasma cells (SLPCs: 7-AAD^–^CD20^–^CD27^+^CD38^+^CD138^–^) and long-lived plasma cells (LLPCs: 7-AAD^–^CD20^–^CD27^+^CD38^+^CD138^+^) were generated from peripheral naive and memory B cells in an in vitro system replicating the bone marrow niche for 6 weeks in a 3-step culture system (Figure 3A; ref. 39). Generation of naive-derived LLPCs and memory-derived LLPCs at day 13 was confirmed to be suboptimal in CVID (*P* = 0.013, and *P* = 0.016, respectively). The deficit was greater in naive-derived LLPCs (7-fold less), while memory-derived LLPCs were only moderately affected (1.5-fold less; Figure 3C). Consistent with the X-inactivation results, our data indicate that suboptimal generation of LLPCs reflects deficits during early differentiation. To investigate whether the reduction in LLPCs was related to a block in phenotypic maturation, we compared the frequencies of persisting SLPCs (CD38^+^CD138^–^) and LLPCs (CD38^+^CD138^+^; Figure 3D). Our data demonstrate largely comparable progressive maturation from CD138^–^ to CD138^+^ plasma cells in both healthy controls and patients with CVID. These data suggest that B cells were ineffective at differentiating into LLPCs because of poor early activation. The deficit was unexpectedly greater in the naive pool.

### Residual LLPCs demonstrate normal functions, suggesting mosaicism within the B cell population.

Because the ability to express the cardinal phenotype of LLPC was partially retained in most patients, we further characterized immunoglobulin production and the expression of key B cell transcription factors. Immunoglobulin production was measured by sandwich ELISA assaying supernatant harvested from the bottom Transwell chambers of the plasma cell cultures. Although IgG and IgA production was significantly lower in patients with CVID compared with healthy controls, IgM outputs by differentiated naive and memory B cells were not significantly different between healthy controls and patients (Supplemental Figure 1A). However, the difference in IgG production between healthy controls and patients was normalized by correcting to the number of plasma cells within the cultures, indicating that the decrease in IgG reflected a decrease in plasma cell number, rather than a functional deficit in secretion among plasma cells. However, the reduction in IgA production could not be attributed to the falling cell count (Supplemental Figure 1B). Consistent with normal plasma cell differentiation, we observed significant upregulation of *BLIMP1*, *XBP1*, and *IRF4* and downregulation of *PAX5* and *BCL6*. Comparable expression was noted between healthy controls and patients with CVID (Supplemental Figure 1C). Our data suggest that the subpopulations of phenotypically normal LLPCs in CVID are functionally competent.

### Altered X chromosome methylation profile after LLPC differentiation.

**Figure 4:**
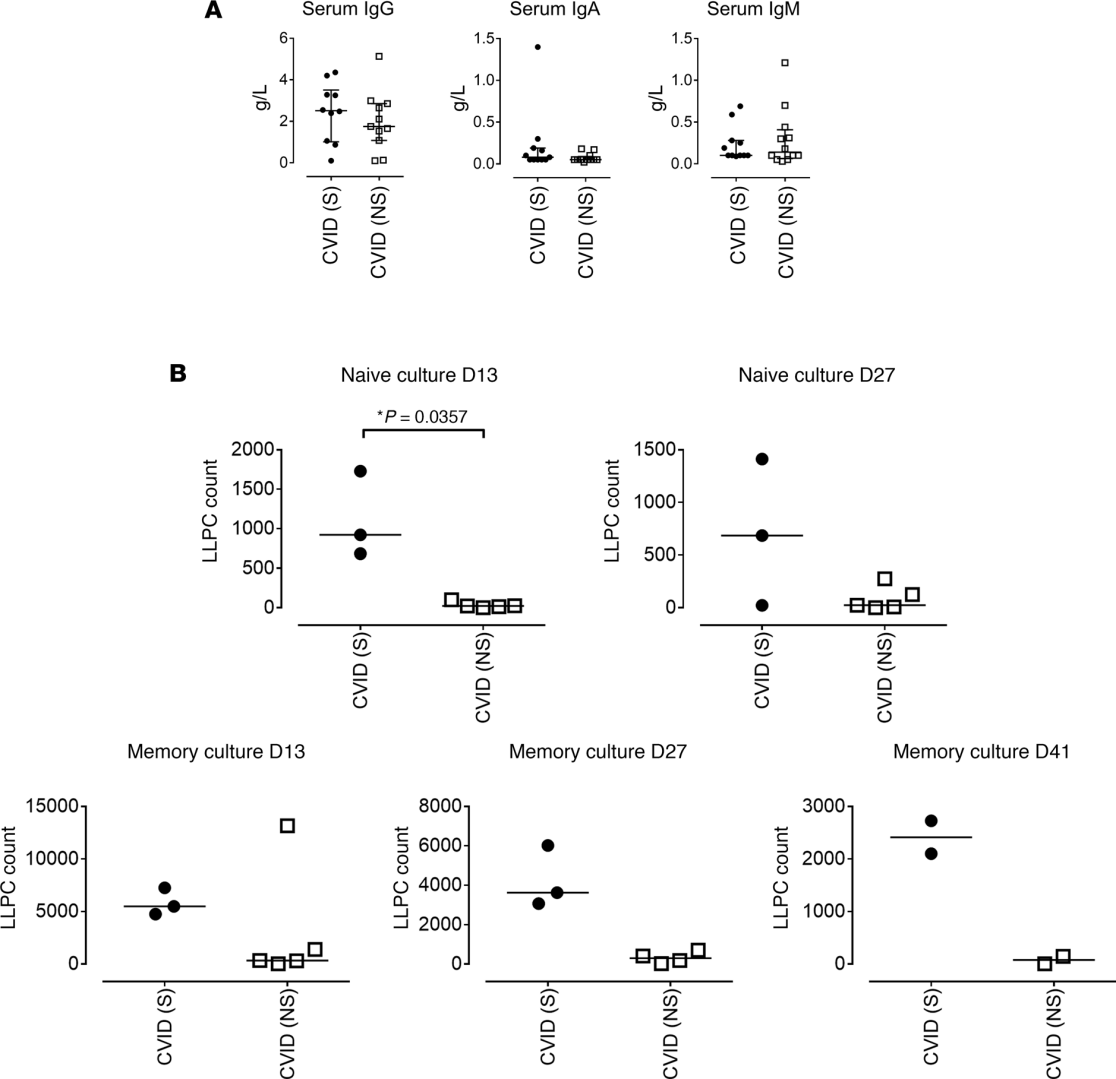
Correlation of X-skewing results with in vitro immunoglobulin production. (**A**) Baseline (before immunoglobulin replacement therapy) serum IgM, IgA, and IgG of X-skewed (S: ≥80% X-skewing, black, *n* = 10) and non–X-skewed patients with CVID (NS: <80% X-skewing, white, *n* = 11) are shown. (**B**) LLPCs: CD20^–^CD27^+^CD38^+^CD138^+^) were generated from isolated naive or memory B cells from X-skewed and non–X-skewed patients with CVID as previously described. LLPC counts within the naive and memory B cell cultures on day 13, day 27, and day 41. Median and interquartile ranges are depicted. Statistically significant differences are highlighted as *P < 0.05 (2-tailed Mann-Whitney *U* test).

To link the findings of abnormal X-skewing and the failure of humoral immunity, we compared the baseline antibody levels and plasma cell–generating ability in patients with and without exaggerated X-skewing (≥80%). Patients with exaggerated X-skewing had higher levels of baseline IgG and IgA, although this did not reach statistical significance (Figure 4A). Furthermore, naive (CD27^–^) and memory (CD27^+^) B cells of patients with at least 80% skewing demonstrated greater ability to generate CD20^–^CD27^+^CD38^+^CD138^+^ LLPCs, with the greatest difference seen from the naive culture on day 13 (*P* = 0.0357; Figure 4B). This suggests that those patients with exaggerated X-skewing may represent a distinct clinical subgroup likely to retain a degree of plasma cell function in comparison with those with complete inert failure of their humoral immunity, although this conclusion is limited because of small sample size.

## Discussion

The clinical and immunophenotypic heterogeneity of patients with CVID has limited a deep understanding of the underlying pathophysiology in the development of the disease. The heterogeneity of presentation and late onset of disease manifestations in many patients with CVID suggest an acquired lymphocyte maturation disorder. By stratifying patients by disease endotypes or immunophenotypes, we may be better able to understand the nuances of CVID manifestations. We have demonstrated clonal hematopoiesis affecting both the myeloid and lymphoid lineages in a subset of patients with adult-onset CVID using DNA methylation as a surrogate marker for bone marrow clonality. The finding of clonality in the blood but not buccal tissue strongly supports that this phenomenon was acquired. Furthermore, our data suggest that the clonally expanded progenitor is likely to have a survival or proliferative advantage over normal hematopoiesis and gradually affects B cell function, while previously differentiated cells continue to contribute to residual LLPC generation and antibody production, although further data in a larger cohort are needed to strengthen this hypothesis. The findings of this study offer an explanation as to why clonality could not be detected using antigen receptor sequencing because this occurs before the VDJ rearrangement (33, 37). Although we have examined only B cell function in this study, future work examining how clonal hematopoiesis could affect other lineages, such as T cells, monocytes, and platelets, would be instructive. Clonal hematopoiesis is known to increase the risk of all hematological malignancies via increasing mutational load (40), although the ultimate event will depend on a number of factors, including further mutation, infection, and cell selection for proliferation and activation. Additionally, although EBV and CMV infections are known to skew B and T cell clonality (41, 42), the presence of X-skewing in both myeloid and lymphoid compartments makes a transient confounding effect from infection unlikely. Future studies evaluating longitudinal samples would be helpful in elucidating these effects further. In keeping with this hypothesis that other cell lineages may be affected, patients with CVID show a greater decline in naive CD4^+^ T cell and B cell numbers with advancing age when compared with the expected physiological rate (43). Furthermore, examining naive and memory T cells could help differentiate clonal origin derived from early myeloid/B cell progenitor to undifferentiated hematopoietic progenitor cells. The phenomenon of clonal hematopoiesis was largely observed in the group 1 patients with CVID (adult-onset patients without noninfectious complications). In contrast, monogenetic causes of CVID are more often detected in group 2 patients with CVID with inflammatory and lymphoproliferative complications, which may be driven by a different mechanism.

Clonal hematopoiesis is relatively rare below the age of 55 years but becomes increasingly common with advancing age (38). It is estimated that close to 20% of people aged more than 90 years may demonstrate clonal hematopoiesis. The initial driver mutations (DNMT3A, TET2, and ASXL1) often result in more aggressive proliferation and antiapoptotic activities, leading to acceleration of physiological mutation rate within the hematopoietic compartment (44). Although the majority of individuals are asymptomatic, clonal hematopoiesis of indeterminate potential (CHIP) has been linked to increased risk of cardiovascular disease, myelodysplasia, hematological malignancies including both myeloid and lymphoid malignancy, acquired aplastic anemia, and paroxysmal nocturnal hemoglobinuria (45–49). In line with this, large CVID cohort studies suggested that there is a 12-fold increase in hematological malignancy, most commonly B cell non-Hodgkin lymphoma (B-NHL; refs. 50–52). This suggests that there may be a link between clonal hematopoiesis, CVID, and B-NHL, where CVID may represent an intermediate stage between CHIP and lymphoma, via increasing genomic instability, drawing parallels between myelodysplasia and acute myeloid leukemia and other malignancies (53, 54). Furthermore, this mechanism may explain the polygenic nature of CVID with limited sharing in nondriver mutations (55). However, myeloid malignancies are relatively infrequent in CVID, and further work is required to understand the bias toward lymphomagenesis.

Certain mutations have been demonstrated to be physiologically crucial for the differentiation of multiple hematological lineages from early hematopoietic development, such as Ikaros family zinc finger protein 1, which has been shown to be a key driver for B cell progenitor acute lymphoblastic leukemia (ALL; refs. 56, 57). Interestingly, in patients with germline heterozygous mutations in Ikaros, with hypogammaglobulinemia with absent B cells, 2 of the 29 patients went on to develop ALL (58). Further studies are needed to evaluate whether a somatic counterpart could be identified in a subset of patients with CVID, explaining both hypogammaglobulinemia and clonal hematopoiesis.

To our knowledge, this study is the first to describe the clonal hematopoiesis in CVID. However, there are limitations to this study. Future studies using high-throughput sequencing with sufficient read depth to characterize variant allele frequency of somatic mutations related to clonal hematopoiesis and other downstream passenger mutations are still needed. A potential technical limitation of the HUMARA assay is the possibility for both over- and underestimation of the degree of X-skewing (59). Nonetheless, the marked difference from the comparatively homogeneous healthy donor population in this study strongly indicates a biological difference between the 2 groups. Future work using additional methylated loci on the X chromosome, such as ZDHHC15, SLITRK4, and PCSK1N, could also strengthen our findings by revealing epigenetic changes in both males and females that are not detectable by the standard genomics approach (60). Although the difference in X-skewing and clonality in group 1 patients with CVID was striking, additional studies with larger numbers of patients and multicenter cohorts are needed to replicate these results and to improve generalizability to a larger population. Additionally, X chromosome inactivation could demonstrate clonality only in female patients, so future studies to demonstrate a similar phenomenon in male patients with CVID are needed, particularly given that sex-specific differences in presentation and clinical outcomes have been reported in patients with CVID (27). Alternative epigenomic approaches, such as ChIP-Seq and DNAse-Seq, would also help elucidate aberrant epigenetic regulation in hematopoietic stem cells. Rodríguez-Cortez and colleagues have investigated epigenetic changes, such as DNA demethylation, and this remains a salient area of research (61–63). Future studies investigating the somatic and epigenetic changes in this disease are needed to determine the cause of the exaggerated X-skewing that was observed predominately in infection-only patients.

The late onset of CVID segregates it from the majority of primary immunodeficiencies. The findings of this study raise the possibility that other adult-onset immunodeficiencies may be triggered by a similar process, especially when somatic mutations can occur at any location across the genome. Consistent with this hypothesis, extreme X-skewing has been reported in carriers of X-linked chronic granulomatous disease and X-linked hyper-IgM patients, who manifest disease later in life (64, 65). Similarly, adult-onset GATA2-deficient patients also demonstrated extreme X-skewing (66). Additionally, this phenomenon was demonstrated by a deep-sequencing approach in other acquired blood disorders, such as paroxysmal nocturnal hemoglobinuria (67). Given that the myeloid compartment was more skewed than the B cell compartment, there may be a common B cell myeloid progenitor. Future work is needed to explore the epigenetic changes in this subgroup of patients and define the true prevalence of this phenomenon.

In summary, this study presents evidence for clonal hematopoiesis in a subset of patients with CVID, supporting a potentially novel mechanism for CVID and offering a unifying explanation for the late-onset, heterogeneous, and premalignant nature of the condition.

## Methods

### Participants.

All patients with CVID were diagnosed according to the ESID-PAGID criteria and had significant reduction in both IgG and IgA. All patients were receiving immunoglobulin replacement therapy. Patients with CVID were subdivided into group 1 (infection only, with or without bronchiectasis) and group 2 (noninfectious complications with polyclonal lymphoproliferation, chronic enteropathy, interstitial lung disease, autoimmunity, or combinations of these).

### Buccal sampling.

Donors were asked to restrict any oral intake for 1 hour before sampling. Distilled water (20 mL) was used to rinse off any residual material immediately before sampling. Donors were asked to take 10 mL of sterile saline into their oral cavity and rub their tongues around the inside of their mouths lightly for about 60 seconds to encourage dislodgement of the surface epithelial layer. The fluid was then recollected into a sterile universal tube for genomic DNA extraction.

### Plasma cell generation.

Plasma cells were generated using a protocol modified from a previous study (39).

Naive and memory B cells were enriched from PBMCs by magnetic bead isolation (human Memory B Cell Isolation Kit, Miltenyi Biotec, or EasySep Human Memory B Cell Isolation Kit, STEMCELL Technologies) according to the manufacturers’ instructions.

Naive and memory B cells were cocultured with irradiated (100 Gy) CD40L-transfected feeder L cells that had been adhered onto a 24-well plate 24 hours earlier. Cultures were grown in Iscove’s modified Dulbecco’s medium (IMDM; MilliporeSigma) supplemented with 10 μg/mL of AffiniPure F(ab′)_2_ fragment goat anti–human IgA + IgG + IgM (H + L) (Stratech, 109-006-064-JIR), 50 ng/mL of IL-21 (eBioscience, 14-8219-80), 20 IU/mL of IL-2 (Proleukin, market authorization number PL31644/0003), 10% fetal calf serum (FCS; MilliporeSigma), and 1% penicillin/streptomycin (MilliporeSigma) for 72 hours at 37°C and 5% CO_2_.

B cells were harvested after 3 days and reseeded onto new wells in IMDM containing 10% FCS, 1% penicillin/streptomycin, 11 μL/mL of HybridoMax (Gentaur), 5 μL/mL of Lipid Mixture (MilliporeSigma), and 20 μL/mL of MEM amino acid solution (MilliporeSigma), supplemented with 50 ng/mL of IL-21 and 20 IU of IL-2, and cultured for a further 72 hours.

On day 5, the M2-10B4 murine fibroblast cells (provided by Reuben Tooze, University of Leeds) were irradiated (80 Gy) and preadhered to the bottom of Transwell chambers (Corning, CLS3398) in IMDM supplemented with 10% FCS and 1% penicillin/streptomycin at a concentration of 4.16 × 10^4^ cells/well.

Naive or memory B cells were harvested and reseeded onto the upper Transwell chambers at 100,000 cells/well on the subsequent day (day 6). The media were supplemented with 50 ng/mL IL-21, 10 ng/mL IL-6 (BioLegend, 570804), and 100 IU IFN-α (Roferon-A).

For long-term culture, the culture media were replaced every 3.5 days with IMDM with FCS, penicillin/streptomycin, HybridoMax, Lipid Mixture, and MEM amino acid solution supplemented with 50 ng/mL IL-21, 10 ng/mL IL-6, and 100 IU IFN-α. IL-21 was withdrawn after day 13. The upper chambers were transferred onto a new 24-well plate with freshly irradiated M210B4 on day 24.

The upper Transwell chambers were harvested at day 13, day 27, and day 41 for flow cytometric analysis.

### Flow cytometric analysis.

Plasma cell cultures were labeled with 1:100 diluted anti–CD20-FITC (BioLegend, 302304), CD27-PE (BioLegend, 302808), CD138-APC (MI15, BioLegend, 356505), and CD38-APC.Cy7 (BioLegend, 303534) on ice for 30 minutes. A human myeloma cell line, U266 (ATCC), was used as a positive control for CD38 and CD138 expression. An isotype control (MOPC-21, BioLegend, 400121) was used to optimize the gating for CD138 (Figure 3B). CountBright Absolute Counting Beads (Life Technologies) were added for accurate cell count. Dead cell stain (7-AAD, BD Pharmingen, 559925) was added before acquisition to exclude dead cells from the analysis. All experiments were performed with the BD Biosciences FACSCanto flow cytometer and analyzed by FlowJo version 7.6.5 software (Tree Star).

### Sandwich ELISA.

Anti–human IgM antibodies (IgM AF6, Serascience, SM100101), anti–human IgA antibodies (IgA MG4.156, Serascience), or anti–human IgG antibodies (IgG R10Z8E9, Serascience, BG100301) were coated onto a NUNC plate (Thermo Fisher Scientific) overnight at 4°C in PBS (1 μg/mL). The wells were washed and blocked with PBS with 10% FCS for 1 hour. The wells were then incubated with standard sera or culture supernatant (1:100 dilution in PBS with 10% FCS) for 2 hours at room temperature. Following another wash, wells were incubated in 1:4,000 of HRP-conjugated goat anti–human κ (SouthernBiotech, 2060-05) plus 1:4,000 of HRP-conjugated goat anti–human λ (SouthernBiotech, 2070-05) for 1 hour at room temperature. One-step Ultra TMB substrate (100 μL, Thermo Fisher Scientific) and 50 μL of 10% hydrochloric acid were used as detection agents according to standard protocol. Light absorbance was measured using the GloMax 96-microplate luminometer.

### Gene expression assay.

Total RNA was extracted from differentiated memory B cells at day 13 using the PureLink RNA Mini Extraction Kit (Life Technologies) according to the manufacturer’s protocol. Total RNA was then converted to cDNA using the high-capacity RNA-to-cDNA Kit (Applied Biosystems). Relative gene expression was measured using the TaqMan gene expression assay (*BLIMP-1*: Hs00153357_m1; *XBP1*: Hs00231936_m1; *IRF4*:Hs01056533_m1; *PAX5*: Hs00277134_m1; and *BCL6*: Hs00153368_m1). The expression averages of *ACTB* (Hs01060665_g1) and *GAPDH* (Hs02758991_g1) were used as endogenous controls, while day 0 naive B cells of the same individuals were used as undifferentiated controls. A final reaction mixture of 20 μL volume was prepared: 1 μL TaqMan probes, 5 μL TaqMan Gene Expression Master Mix (Applied Biosystems), and 30 ng cDNA in nuclease-free water. Relative fluorescence intensity was measured using the 7900HT Fast Real-Time PCR System (Applied Biosystems). Relative gene expression was calculated by correcting to the endogenous and undifferentiated controls: ΔΔCT = (CT [target, untreated] – CT [reference, untreated]) – (CT [target, treated] – CT [reference, treated]); ratio = 2^ΔΔCT^.

### X-inactivation assay.

The methylation profile of the AR gene, located on the X chromosome, was used as a surrogate marker for bone marrow clonality. X chromosome skewing ratios were determined by the HUMARA assay. Genomic DNA was extracted from whole blood, PBMCs, granulocytes, enriched naive B cells, and memory B cells using the PureLink Genomic DNA Kits (Life Technologies) according to the standard manufacturer’s protocol. Granulocytes were isolated as previously described (68).

### Restriction enzyme digestion.

Undigested DNA (Reaction A) and restriction enzyme–digested DNA (Reaction B) were prepared from each individual. Reaction A contained 50 ng genomic DNA, 2 μL Buffer A (Promega), 0.2 μL of 10 mg/mL acetylated bovine serum albumin (BSA; Promega), and nuclease-free water to a final volume of 20 μL. Reaction B contained 50 ng genomic DNA, 0.5 μL HpaII restriction enzyme (Promega), 2 μL Buffer A (Promega), 0.2 μL acetylated BSA (Promega), and molecular-grade water to a final volume of 20 μL. The reactions were incubated in a thermocycler at 37°C for 2 hours to destroy the unmethylated AR allele.

### PCR amplification of human AR gene.

PCR was performed using the AccuPrime Taq DNA Polymerase High Fidelity Kit (Invitrogen) as follows: 5 μL digested or undigested DNA, 5 μL 10× AccuPrime PCR Buffer II, 0.2 μL AccuPrime Taq High Fidelity, 37.8 μL molecular-grade water, 1 μL of 10 μmol/L fluorescein amidite–labeled (FAM-labeled) forward primer (AR-F: FAM 5′-TCCAGAATCTGTTCCAGAGCGTGC-3′), and 1 μL of 10 μmol/L reverse primer (AR-R: 5′-GCTGTGAAGGTTGCTGTTCCTCAT-3′). PCR was conducted as follows: step 1, 94°C for 30 seconds; step 2, 94°C for 30 seconds; step 3, 56°C for 30 seconds; step 4, 68°C for 60 seconds; step 5, repeat steps 2, 3, and 4 for a total of 34 cycles; and step 6, 4°C holding.

### Capillary reaction.

Following PCR, reactions were diluted to 1:50 in molecular-grade water. In a 96-well PCR plate, 1 μL of the diluted reaction was added to a mixture containing 8.5 μL of Hi-Di formamide and 0.5 μL of GeneScan 600 LIZ dye Size Standard v2.0 (Life Technologies). The PCR products were denatured by a further cycle at 95°C for 5 minutes. Analysis was carried out using an ABI 3730 DNA analyzer (Applied Biosystems). The data were analyzed on Gene Mapper version 4.0. X chromosome skewing was estimated as previously described (69). The germline adjusted ratio was calculated by dividing X-skewing of the lymphocyte subset by X-skewing of the buccal tissue.

### Statistics.

Statistical analyses were performed using GraphPad Prism version 5.0. Comparison between groups was performed using the Mann-Whitney *U* test. Two-way ANOVA was used when multiple groups were analyzed. A *P* value of less than 0.05 was considered significant.

### Study approval.

The study was conducted according to protocols approved by the South Birmingham Research Ethics Committee, Redditch, United Kingdom (REC reference 11/WM/0041). All participants provided written informed consent in accordance with the Declaration of Helsinki.

## Author contributions

GKW designed the study and performed research, analyzed data, and wrote the paper. SB and JMH analyzed data and wrote the paper. DM and SAP performed research. AH provided and analyzed clinical data. AR designed the study and critically reviewed the manuscript. MC designed the study, analyzed data, and wrote the paper.

## Supplementary Material

Supplemental data

## Figures and Tables

**Table 1:**
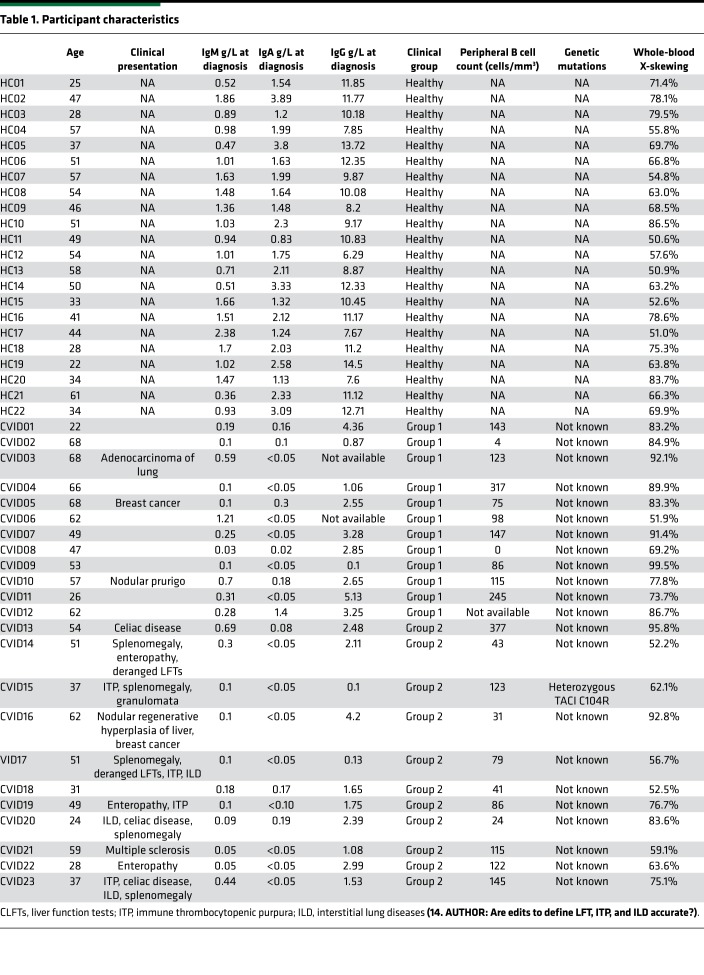
Participant characteristics

## Version 1 07/25/2019

Electronic publication
