# Supplementary material for: Aberrant X chromosome skewing and acquired clonal hematopoiesis in adult-onset common variable immunodeficiency
Source: JCI Insight. 2019 Jul 25;4(14):e127614. doi: 10.1172/jci.insight.127614 (PMC6675553; doi:10.1172/jci.insight.127614)
Supplement: Supplemental data [file jciinsight-4-127614-s222.pdf]

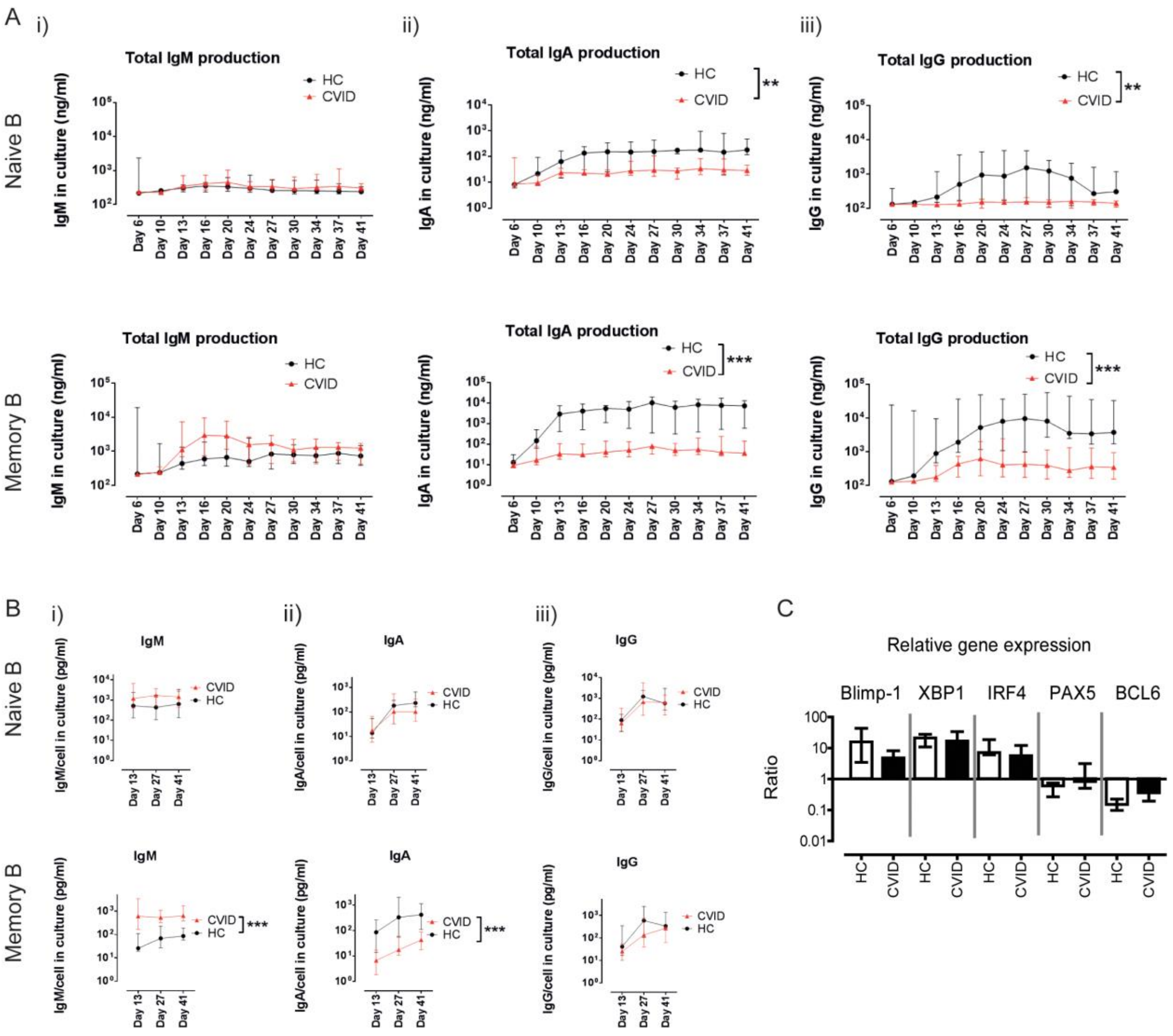

Fig. S1. Subpopulation of phenotypically normal LLPCs in CVID exhibit normal functions. Supernatant was collected from the bottom Transwell chambers of plasma cell cultures between Day 6 to Day 41, every 3.5 days. Sandwiched ELISA was used to measure the total amounts of IgM, IgA, and IgG in the cultures. IgM, IgA, and IgG were corrected to the total plasma cell counts (SLPC + LLPC) on Day 13, Day 27, and Day 41 (HC, n=10-6; CVID, n=14-8). Statistical differences were calculated by two-way ANOVA. Median and interquartile ranges are shown (A-B). Plasma cell cultures were harvested on Day 13 and washed with PBS before total mRNA was extracted for qPCR. Gene expression ratios ($2^{\Delta\Delta CT}$) of *BLIMP-1*, *XBP1*, *IRF4*, *PAX5*, and *BCL6* were calculated using endogenous (average of beta-actin and GAPDH) and undifferentiated controls (Day 0 naïve B-cells). Statistical differences between HC (n=5) and CVID (n=5) were calculated by Mann-Whitney U test. Median and interquartile ranges are shown (D). Statistical significances are indicated as *p<0.05, **p<0.01, and ***p<0.001.

Supplementary Table:

Supplementary Table 1. Clinical presentation of patients demonstrating clonal hematopoiesis.

Patient no.	Age of presentation	IgM g/L	IgA g/L	IgG g/L	Bronchiectasis	Splenomegaly, lymphadenopathy	Autoimmune, inflammatory complications	Hb g/L
C1	43	0.59	<0.05	Historical	Mild	N	N	132
C2	59	<0.10	<0.05	1.06	Mild	N	N	165
C3	37	0.25	<0.05	3.28	Mild	N	N	128
C4	29	0.69	0.08	2.48	Mild	N	N (Coeliac)	128
C5	13	0.31	<0.05	5.13	Mild	N	N	123
	**WBC 10^9/L**	**Neutrophils 10^9/L**	**Platelet 10^9/L**	**CD3 cells/mm^3**	**CD4 cells/mm^3**	**CD8 cells/mm^3**	**CD19 cells/mm^3**	**NK cells/mm^3**
C1	4.47	3.15	217	596	452	108	123	241
C2	9.41	6.89	524	1000	607	321	317	317
C3	5.13	3.38	381	658	446	193	147	301
C4	8.43	5.16	359	1008	577	453	377	19
C5	6.75	3.64	318	1517	598	848	245	337
